# ^1^H NMR Combined with Multivariate Statistics for Discrimination of Female and Male Flower Buds of *Populus tomentosa*

**DOI:** 10.3390/molecules26216458

**Published:** 2021-10-26

**Authors:** Bo Xu, Cui Wu, Zhuojun Li, Pingping Song, Zhimao Chao

**Affiliations:** Institute of Chinese Materia Medica, China Academy of Chinese Medical Sciences, Beijing 100700, China; xubo_345@163.com (B.X.); wucuidalian@163.com (C.W.); 18811385399@163.com (Z.L.); songpingping122@163.com (P.S.)

**Keywords:** Chinese medicine, principal component analysis, hierarchical clustering analysis, orthogonal partial least squares-discriminant analysis, dioecious plants

## Abstract

^1^H Nuclear Magnetic Resonance (^1^H NMR) combined with multivariate statistics was adopted to discriminate female and male flower buds of *Populus tomentosa* in the study. Samples of 11 female and 16 male flower buds of *P. tomentosa* were collected in Beijing, China. ^1^H NMR spectra were acquired on a 400 MHz spectrometer. In total, 30 chemical compounds were identified with standards and literature according to chemical shifts, peak areas, and multiplicity. Principal component analysis (PCA), hierarchical clustering analysis (HCA), and supervised orthogonal partial least squares-discriminant analysis (OPLS-DA) were applied to discriminate female and male flower buds. An apparent grouping trend (R^2^X, 0.809; Q^2^, 0.903) between female and male groups was exhibited with PCA and HCA. The two groups were also well discriminated with OPLS-DA (R^2^X, 0.808; R^2^Y, 0.976; Q^2^, 0.960). Combined with variable importance in projection (VIP) > 1.0 and *p <* 0.05 of OPLS-DA, it was found that the content of daucosterol, *β*-sitosterol, ursolic acid, and betulonic acid in male group was higher than that in female, which should be the key differences of chemical constituents in female and male flower buds of *P. tomentosa*. The study demonstrated that ^1^H NMR combined with multivariate statistics could be used to discriminate female and male plants and clarify differences, which provided a novel method to identify the gender of dioecious plants.

## 1. Introduction

Dioecious plants refer to the plants that have female and male flowers grown in different individuals. Although the incidence of dioecy in flowering plants globally is relatively low (6~7%) [1], there are several traditional Chinese medicines from dioecious plants, such as Duzhong (Eucommiae Cortex) from *Eucommia ulmoids* Oliv. (Fam. Eucommiaceae), Yinxingye (Ginkgo folium) from *Ginkgo biloba* L. (Fam. Bilobaceae), and Tianhuafen (Trichosanthis Radic) from *Trichosanthes kirilowii* Maxim. and *T. rosthornii* Harms (Fam. Cucurbitaceae) [2]. Most traditional Chinese medicines derived from dioecious plants are applied in clinical and pharmaceutical practice regardless of their gender.

*Populus tomentosa* Carrière (Fam. Salicaceae), a typical kind of dioecious plant, is widely distributed in North China Plain [3] and planted by the roadside to provide shelterbelts due to their strong adaptability, rapid growth, cooling [4], noise reduction [5], increasing air humidity [6], and reinforcing soil [7]. The dried male inflorescence of *P. tomentosa*, called Yangshuhua in Chinese, has been applied in clinical practice for bacillary dysentery and acute enteritis in Pharmacopoeia of the People’s Republic of China [8]. Studies have shown that the aqueous extract of male inflorescence of *P. tomentosa* possessed anti-inflammatory, anti-diarrheal, anti-microbial, and analgesic activities [9,10]. The fresh male inflorescence is used as food materials in some places of China. However, the female inflorescence is neither included in Chinese pharmacopoeia nor adopted as food materials. Therefore, it is of great significance to discriminate female and male inflorescence. Inflorescence is developed from flower buds and identifying the female and male flower buds can help knowing the gender of inflorescence in advance. But the female and male flower buds of *P.* *tomentosa* are highly similar in appearance and can’t be distinguished visually. In addition, the difference of chemical compositions in flower buds, one of reproductive organs, can better reflect the characteristics of genders. Therefore, it is necessary to establish an analytical method to discriminate them and filter the differences in chemical compositions.

At present, some studies have been carried out on female and male *P. tomentosa.* For example, 13 chemical constituents were isolated and identified from male bark of *P. tomentosa*, including sakuranin, salicyltomenside, and siebolside B [11,12]. The content of micranthoside, siebolside B, sakuranin, and isosakuranin was different in female and male barks of *P. tomentosa* by high-performance liquid chromatography (HPLC) fingerprint method with multivariate statistical analyses [13]. Volatile components in female and male flower buds were compared with HS-SPME-GC-MS (headspace-solid phase microextraction-gas chromatography-mass spectrometer). It was found that the content of 2-cyclohexen-1-one, benzyl benzoate, and methyl benzoate in female was significantly higher than that in male, and the content of ethyl benzoate in male was significantly higher than that in female [14]. However, the differences of non-volatile components between female and male flower buds of *P. tomentosa* have not been reported yet.

HPLC, liquid chromatography-mass spectrometry (LC-MS), and GC-MS usually require chromatographic separation of chemical compositions before detection, whereas ^1^H NMR can be applied directly without prior chromatographic separation. Therefore, chemical compositions can be reflected more comprehensively with ^1^H NMR. Besides, the application of GC-MS is limited by the volatility of analytes. For non-volatile compounds, they should be processed by derivatization reaction, which is unstable, time-consuming, and complex. ^1^H NMR can detect a variety of chemical compositions at low concentrations in non-destructive manner without complex pre-treatment [15,16,17]. It shows the signal peaks of all protons as a whole and areas of signal peaks are proportional to the number of protons and relative concentrations in samples. Therefore, it can be applied in both qualitative and quantitative analysis. It has become a useful discrimination tool in recent years, such as the detection of dairy food fraud [18], adulteration of herbal medicines [19], and classification of wine varieties [20]. At present, ^1^H NMR has been widely applied in medical care [21,22], food [23,24,25], chemistry [26], and other fields.

In order to discriminate female and male flower buds of *P. tomentosa* and clarify the differences in chemical compositions, 11 female and 16 male flower buds of mature *P. tomentosa* were collected in Beijing, China. ^1^H NMR combined with PCA (principal component analysis), HCA (hierarchical clustering analysis), and OPLS-DA (orthogonal partial least squares-discriminant analysis) were applied. With variable importance in projection (VIP) > 1.0 and *P <* 0.05 of OPLS-DA, differences of chemical compositions were screened in the study.

## 2. Results

### 2.1. Visual Inspection of ^1^H NMR Spectra

**Figure 1:**
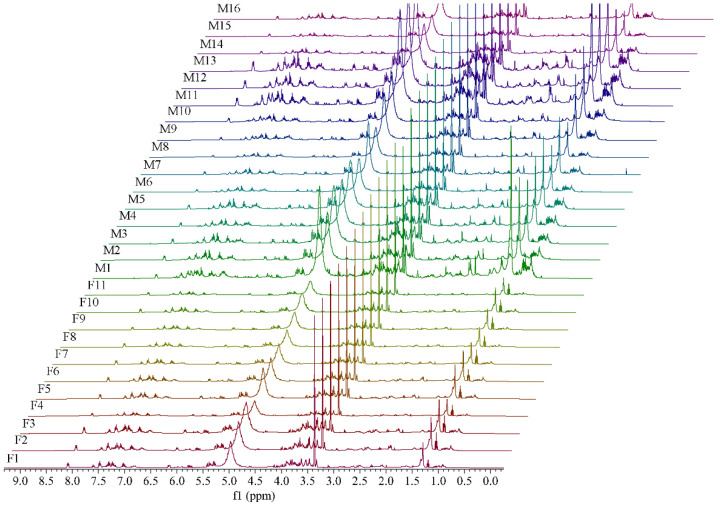
^1^H NMR spectra of 11 female and 16 male flower buds of *P. tomentosa* with the same amplification degree.

**Figure 2:**
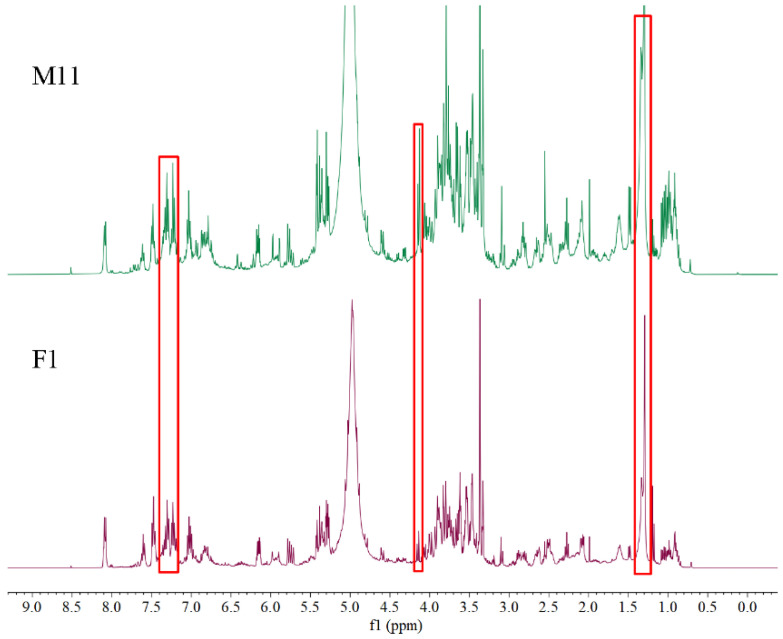
Representative ^1^H NMR spectra of female (F1) and male (M11) flower buds of *P. tomentosa* with the same amplification degree.

^1^H NMR spectra of 11 female samples and 16 male samples are shown in Figure 1. The representative ^1^H NMR spectra of female (F1) and male (M11) samples with the same amplification degree are shown in Figure 2. It can be observed that female and male samples have similar profile on a whole and several differences in intensities of peaks. Some obvious differences by visual inspection are highlighted with red boxes in Figure 2. For example, the intensities at the chemical shift of 1.30 (unknown), 4.13 (sucrose), 7.23 (myricetin), and 7.31 (caffeic acid) in M11 are higher than those in F1, which indicates that there exist differences in content of chemical compositions between female and male flower buds of *P. tomentosa*.

### 2.2. Compound Assignment

**Figure 3:**
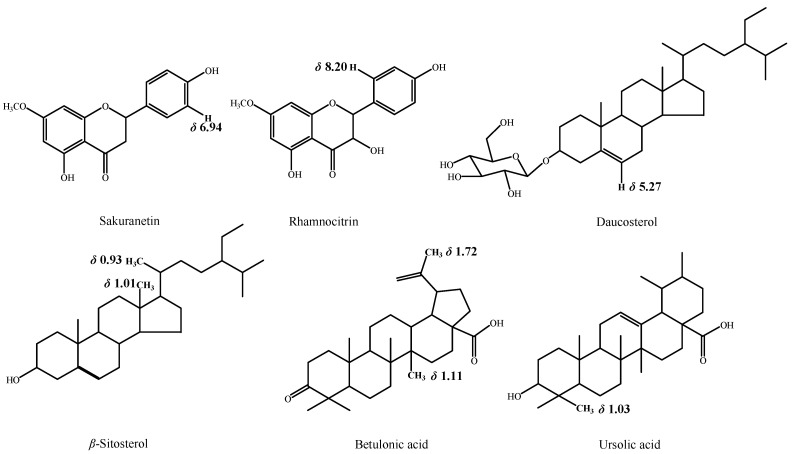
Some chemical structures with the specific hydrogen atoms corresponding to the mentioned chemical shifts.

According to standards and literature [11,27,28,29,30], combined with chemical shifts, peak areas, and multiplicity, 30 compounds are identified and summarized in Table 1. Some chemical structures are displayed in Figure 3 together with the specific hydrogen atoms corresponding to the mentioned chemical shifts in Table 1.

### 2.3. PCA and HCA

**Figure 4:**
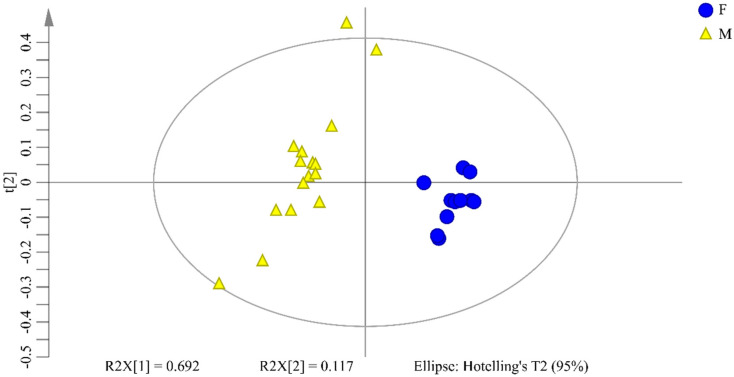
PCA scores plot for female and male flower buds of *P. tomentosa*. Circles represent female samples and triangles represent male samples. The X axis refers to the score of the first principal component (PC1) and the Y axis refers to the score of the second principal component (PC2). The variances accounted by PC1 and PC2 are 69.2% and 11.7%, respectively.

PCA is performed to evaluate the differences between female and male groups. The result of PCA score plots is displayed in Figure 4. It can be clearly observed a clear grouping trend between female and male groups, which indicates female and male flower buds of *P. tomentosa*, can be discriminated with PCA. The first contribution value of two principal components is accounted for 80.9% (R^2^X) in the total variance (PC1 for the first principal component described 69.2% and PC2 for the second principal component described 11.7% of the sample variability). The predictive ability of the model (Q^2^) is 90.3%, which demonstrates that it is a good model.

**Figure 5:**
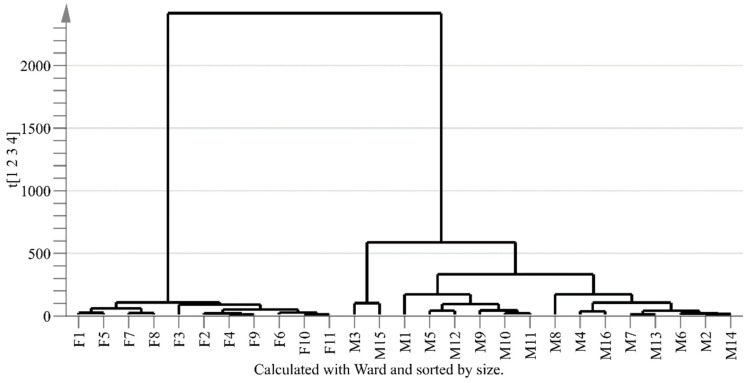
Dendrogram of HCA. F1~F11 refer to the female samples and M1~M16 refer to the male samples. The X axis refers to sample name and Y axis refers to the distances between different groups.

HCA is carried out based on the first four PCs from the PCA model, and displays relationships between female and male groups in the form of dendrogram. As shown in Figure 5, 27 samples could be clearly separated into two groups, i.e., all of the 11 female samples are classified into Group 1 (left) and 16 male samples are classified into Group 2 (right). Though the distribution of some male samples in score plot of PCA is dispersed, they are clearly divided into group 1 in the dendrogram of HCA. The result of HCA confirms the classification with PCA model. These results demonstrate that there are differences in chemical compositions between female and male flower buds of *P. tomentosa*.

### 2.4. OPLS-DA

**Figure 6:**
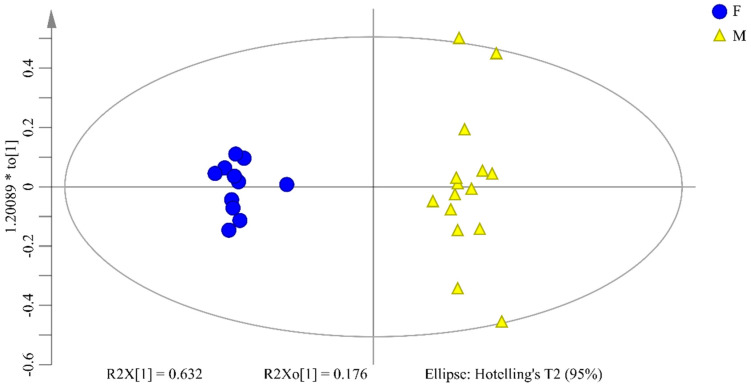
OPLS-DA scores plot for female and male flower buds of *P. tomentosa*. Circles represent female samples and triangles represent male samples. The X axis refers to the score of the first principal component (PC1) and the Y axis refers to the score of the second principal component (PC2). The variances accounted by PC1 and PC2 are 63.2% and 17.6%, respectively.

OPLS-DA is performed to explore the chemical composition differences between female and male flower buds of *P. tomentosa*. The score plot is shown in Figure 6. R^2^X of OPLS-DA model is 80.8%, demonstrating that 80.8% of variation can be modeled by the selected components. R^2^Y is 97.6%, indicating that the model is well fitted. Q^2^ is 96.0%, demonstrating that it has a good predictability.

With VIP > 1.0 and *p* < 0.05 to filter the differences that are responsible for differentiating female and male groups, it is found that areas of *δ* 0.80~2.00 and *δ* 6.96~8.08 in ^1^H NMR spectra of male group are significantly higher than those of female. In other regions, there is not an apparent difference between the two groups. Furthermore, combined with the 30 compounds and the paired Student’s *t*-test for the significance analysis, the content of daucosterol, *β*-sitosterol, ursolic acid, and betulonic acid in male group is higher than that in female group (*p* < 0.05). It can be concluded that the unequal content of these compounds should be the key differences of chemical constituents in female and male flower buds of *P. tomentosa*.

## 3. Materials and Methods

### 3.1. Reagents

Methanol was of analytical grade and purchased from Tianjin Fuyu Fine Chemical Co., Ltd. (Tianjin, China). Methanol-d_4_ (CD_3_OD, 99.8%) with 0.03% trimethylsilyl (TMS) was purchased from Sigma-Aldrich, Inc. (St. Louis, MO, USA).

### 3.2. Apparatus

A DFT-50A grinder was purchased from Wenling Linda Machinery Co., Ltd. (Wenling, China). An Ohaus CP224C electronic balance was purchased from Ohaus (Shanghai) Instrument Co., Ltd. (Shanghai, China). A KQ-100E ultrasonic instrument was purchased from Kunshan Ultrasonic Instrument Co., Ltd. (Kunshan, China). ^1^H NMR spectra were acquired on a Brucker AVANCE II 400 MHz spectrometer (Brucker, Bremerhaven, Germany).

### 3.3. Sample Collection

Samples of 11 female (F1~F11) and 16 male (M1~M16) flower buds of *P. tomentosa* were collected on 23~28th, February, 2020 in Beijing, China. The detailed sample information is shown in Table 2. The gender of all samples was clearly identified on 6th, April, 2020 based on their mature flowers and/or fruits.

### 3.4. Sample Preparation

Samples were dried in shade and ventilated environment for 10 days with temperature of 18 ± 5 °C and relative humidity of 25~40%, crushed into powder with a grinder, and passed through a 20-mesh sieve. Powder sample of 1.0 g was accurately weighed into 50 mL conical flask and 25 mL of methanol was added precisely. Samples were weighed, ultrasonically extracted for 30 min (100 W, 40 kHz), and weighed again after cooling to room temperature. Weight losses of extracts were replaced with methanol. The obtained extracts then were filtered and filtrates were placed into 35 mL evaporating dishes and concentrated at room temperature. 1.50 mL CD_3_OD with 0.03% TMS was added, dissolved ultrasonically, and filtered with 0.22 μm membrane filter. A liquor of 0.8 mL was transferred into nuclear magnetic tube for analysis.

### 3.5. ^1^H NMR Measurement

The ^1^H NMR spectra were acquired on a Bruker AVANCE II 400 MHz spectrometer at 293 K. TMS and CD_3_OD provided chemical shift reference (^1^H, *δ* 0.00) and field frequency lock, respectively. A zg30 pulse sequence was used to suppress the residual H_2_O signal. A total of 16 transients were collected using a spectral width of 8012 Hz with acquisition time of 4.09 s and relaxation delay of 1.00 s.

### 3.6. Data Processing

#### 3.6.1. Spectra Pre-Treatment

Free induction decay files were imported into MestRenova software (Version 14.0, Mestrelab Research SL, Santiago de Compostela, Spain). Automatic baseline correction and manual phase correction were performed. The proton signal of TMS was calibrated to *δ* 0.00. ^1^H NMR spectra were integrated into bins with width range of 0.04 ppm. The total integration width was *δ* 0.00~9.00 except for the regions of *δ* 3.31~3.35 corresponding to methanol and *δ* 4.70~5.10 for residual water. Signal areas of bins were normalized to the sum of spectra to compensate for the differences in concentrations and acted as variable values for further statistical analysis [31,32].

To investigate the repeatability of the method, six replicates of both F1 and M11 were evaluated, respectively. The results were expressed with correlation coefficient [33]. The correlation coefficients of both F1 and M11 samples were higher than 0.99, respectively, which indicated that the repeatability was good.

#### 3.6.2. Statistical Analysis

All multivariate analysis and calculations were performed on SIMCA-P software (version 14.1, Umetrics, Malmö, Sweden). The data were imported into the software and scaled by Pareto scaling method to reduce the relative importance of large values and to keep the data structure partially intact [34]. Then the data were submitted to PCA, HCA, and OPLS-DA analysis, respectively [35,36].

PCA is an unsupervised statistical method for reducing dimensions of a database by linear combinations of a starting set of variables based on their maximum variance [37] and can convert original variables into new independent variables named principal components (PCs) [7]. It can make a preliminary judgement on the distribution status, natural aggregation, and abnormal samples [38]. R^2^ and Q^2^ are the important metrics to evaluate PCA model. R^2^ indicates the ability of PCs to explain the variation in a variable and Q^2^ is the predictability of the model. R^2^ and Q^2^ closing to 1.0 demonstrates that the model is reliable and has good fitting accuracy [36]. HCA was also carried out by SIMCA-P software with the first four principal components of PCA model. The distances among samples were calculated through Ward’s minimum-variance method. The result was presented as a dendrogram in the study.

OPLS-DA extends a regression of PCA, uses the class membership to maximize the variation, and introduces an orthogonal signal correction filter to separately handle the systematic variation correlated or uncorrelated to the *Y* variable. Therefore, it has better discriminant ability for the samples with larger within-class divergence than that of PCA [39]. R^2^X, R^2^Y, and Q^2^ are the parameters frequently used to evaluate OPLS-DA model. R^2^X shows the capability of differentiating groups in established model. R^2^Y and Q^2^ reflect the goodness-of-fit and ability of prediction in models, respectively. Values of the three parameters closing to 1.0 indicate reasonably good differentiating, fitness, and prediction for the constructed model [40].

To further explore the chemical compositions that contribute significantly for the sample discrimination, VIP scores were adopted. VIP scores larger than 1.0 demonstrates that variables are important for sample discrimination. In this study, VIP > 1.0 and *P* < 0.05 were used to select variables that contribute greatly to the differences between female and male groups [41]. The paired Student’s *t*-test was performed for the significance analysis.

### 3.7. Chemical Compound Assignment

The chemical compounds were identified by adding standards or consulting literature according to chemical shifts, peak areas, and multiplicity. Standards considered in the present samples were added. The presence of added standards was confirmed if the existing peaks in the spectra increased, whereas their presence was ruled out if new peaks appeared.

## 4. Discussion

There are no significant differences in ^1^H NMR spectra of the 11 female samples visually, nor are the 16 male samples. The female and male samples have similar profile in chemical shifts and multiplicity but several differences in intensities of peaks, which implies that the content of related compounds is different. In addition, 30 compounds are assigned with ^1^H NMR spectra. All of the compounds are the secondary metabolites of plants. The 27 samples could be divided into two groups with PCA, HCA, and OPLS-DA analysis successfully, which are coincident with their gender. The result proves that ^1^H NMR combined with multivariate statistics to discriminate gender is feasible, which provides a novel idea for the classification of dioecious plants.

The content of compounds could be reflected with related intensities of ^1^H NMR spectra. Based on this principle, it is found that the content of four compounds, i.e., daucosterol, *β*-sitosterol, ursolic acid, and betulonic acid, in male group are significantly higher than that in female group. All of the four compounds have notable pharmaceutical activities. The effects of daucosterol have been reported for the suppression of cancer, promotion of neural stem cell proliferation, induction of Th1 immune response, and especially suppression of acute enteritis [42]. As mentioned in introduction, only the male was applied in clinical practice for bacillary dysentery and acute enteritis. This study shows that daucosterol is one of markers to distinguish the female and male flower buds of *P. tomentosa*. Furthermore, its higher content in male samples may be the reason why the male is adopted for acute enteritis. The pharmacological activities of ursolic acid are antimicrobial activity [43], antivirus [44], anticancer [45], vascular protection [46], and Gram-positive bacteria inhibition [44]. The antimicrobial activity and high content of ursolic acid in male may play an important role for the male to treat bacillary dysentery and acute enteritis, which are mainly caused by bacterial infection.

*β*-Sitosterol can regulate the tumor growth by decreasing the membrane fluidity, reduce the fluidity of fatty acyl group of phospholipids, increase the thickness of double layer coating of lipidosome, and maintain the stability of lipidosome [47]. It is reported that betulonic acid is a bioactive substance exhibiting antiviral, cytotoxic, and antiangiogenic activities. It is also widely used as an intermediate in synthesis of various triterpenoid derivatives with anti-inflammatory, antiviral, and antiproliferative properties [48]. The anti-inflammatory activity of *β*-sitosterol and betulonic acid with high content in male may be conducive to the treatment of bacillary dysentery and acute enteritis. In summary, the four compounds with higher content are not only markers for distinguishing the female and male, but also accounted for the application of the male in clinical and pharmaceutical practice.

## 5. Conclusions

In this study, ^1^H NMR combined with multivariate statistics was used to study differences in chemical compositions between female and male flower buds of *P. tomentosa*. Several differences in intensities of signals could be observed by visual inspection. The female and male groups were clearly differentiated with PCA, HCA, and OPLS-DA analysis. Furthermore, to discover the differences in chemical compositions between them, 30 compounds were assigned with standards and literature. Four compounds, i.e., daucosterol, *β*-sitosterol, ursolic acid, and betulonic acid were filtered as markers for their higher content in male samples. The pharmaceutical activities of the four markers were coincident with their clinical practice of the male. It is the first time that differences in chemical composition of the dioecious plant have been revealed with ^1^H NMR, which reveals the essential difference in secondary metabolites of dioecious plants.

## Figures and Tables

**Table 1:** The assigned compounds with ^1^H NMR spectra (s, singlet; d, doublet; and t, triplet).

No.	Compound Name	*δ*_H_ (Multiplicity, Coupling Constant, Protons)	References
1	Benzoic acid	7.60 (t, 7.6, 1H), 7.48 (t, 7.6, 2H)	[11,27]
2	Sakuranetin	6.94 (d, 8.0, 2H)	Standard and [11]
3	Rhamnocitrin	8.20 (d, 9.2, 2H)	[11]
4	Salicyltremuloidin	7.35 (t, 8.0, 2H), 7.25 (d, 8.0, 1H)	[11]
5	Daucosterol	5.27(d, 4.8, 1H), 0.96 (s, 3H)	[11,28,29]
6	Tremuloidin	7.30 (d, 7.6, 1H)	[11]
7	Isograndidentatin A	6.84 (d, 8.4, 2H), 4.51 (d, 8.0, 1H)	Standard and [11]
8	Siebolside B	5.53 (d, 13.2, 1H)	Standard and [11]
9	Sakuranin	3.79 (s, 3H)	[11]
10	Micranthoside	7.34 (d, 7.6, 2H)	[11]
11	Sucrose	5.42 (d, 3.6, 1H), 4.13 (d, 8.4, 1H)	[11]
12	*β*-Sitosterol	1.01 (s, 3H), 0.93 (d, 6.4, 3H), 0.81 (d, 8.4, 3H)	[11]
13	Salicin	7.19 (d, 7.2, 1H)	[11,27,29]
14	Eriodictyol	6.83 (s, 1H), 6.78 (s, 2H)	[28]
15	Myricetin	7.23 (s, 2H)	[28]
16	Dihydromyricetin	6.41 (s, 2H), 5.94 (d, 2.0, 1H)	[28]
17	Protocatechuic acid	7.33 (d, 1.6, 1H)	[28]
18	Caffeic acid	7.31 (d, 15.2, 1H)	[28]
19	2-Acetyl-1,3-dicaffeoylglycerol	2.06 (s, 3H)	[28]
20	Quercetin	6.82 (d, 8.8, 1H)	[28]
21	Betulonic acid	1.72 (s, 3H), 1.11 (s, 3H)	[30]
22	Chrysoeriol	7.57 (d, 2.0, 1H)	[30]
23	Pinocembrin	7.49(d, 8.0, 2H), 5.97 (d, 2.4, 1H)	[30]
24	Dillenetin	7.14 (d, 8.8, 1H)	[30]
25	Naringenin	7.32 (d, 8.0, 2H)	[30]
26	Isosakuranetin	6.97 (d, 8.8, 2H)	[30]
27	Apigenin	8.00 (d, 9.2, 2H)	[30]
28	Kaempferol	6.42 (d, 2.0, 1H), 6.22 (d, 2.0, 1H)	[30]
29	3,3′,4,4′-Tetrahydroxybiphenyl	6.81 (d, 2.0, 2H)	[30]
30	Ursolic acid	1.03 (s, 3H)	[30]

**Table 2:** Collection locations of samples in Beijing, China.

No.	Collection Location	Longitude and Latitude
F1~F5	Qing Long Hutong	39°56′53.74″ N, 116°25′24.82″ E
F6~F8	Min’an Community	39°56′35.12″ N, 116°25′49.62″ E
F9~F11	Lishuiqiao South	40°13′14.77″ N, 116°13′52.61″ E
M1~M2	Bei’erhuan Road	39°56′56.79″ N, 116°25′20.40″ E
M3~M10	Tongjiao Temple	40°13′14.77″ N, 116°13′52.61″ E
M11~M16	Min’an Street	39°56′40.67″ N, 116°25′48.47″ E

All *P. tomentosa* for sample collection were in adulthood. The truck heights of all *P. tomentosa* were more than 10 m. The circumferences at a height of 1.5 m were between 85 to 197 cm. All samples were identified as female or male flower buds of *Populus tomentosa* Carrière (Fam. Salicaceae) by Prof. Zhimao Chao (Institute of Chinese Materia Medica, China Academy of Chinese Medical Sciences) according to the description in Flora of China (Editorial Board of Flora of China, 1984). The voucher specimens were deposited at 1022 laboratory of Institute of Chinese Materia Medica, China Academy of Chinese Medical Sciences, Beijing, China.

## Data Availability

All the relevant data have been provided in the manuscript. The authors will provide additional details if required.

## Sample Availability

Not available.

## Abbreviations

^1^H NMR: ^1^H Nuclear Magnetic Resonance; PCA, principal component analysis; HCA, hierarchical clustering analysis; OPLS-DA, orthogonal partial least squares-discriminant analysis; VIP, variable importance in projection; HPLC, high-performance liquid chromatography; HS-SPME-GC-MS, headspace-solid phase microextraction-gas chromatography-mass spectrometer; LC-MS, liquid chromatography-mass spectrometry; TMS, trimethylsilyl, PCs: principal components.
